# Potentially Inappropriate Medication Use in Older Adults: Concordance Among the Chinese and AGS/Beers and STOPP Criteria

**DOI:** 10.3390/jcm15155826

**Published:** 2026-07-25

**Authors:** Zhaoyan Chen, Fangyuan Tian, Xuehua Wan, Fei Zhou, Fengbo Wu

**Affiliations:** 1 Department of Pharmacy, National Clinical Research Center for Geriatrics, West China Hospital, Sichuan University, Chengdu 610041, China; 2 Department of Epidemiology and Health Statistics, West China School of Public Health, West China Fourth Hospital, Sichuan University, Chengdu 610041, China

**Keywords:** potentially inappropriate medications, older, criteria, outpatient, risk factors

## Abstract

**Objectives**: The aging population in China presents significant challenges related to polypharmacy and potentially inappropriate medication (PIM) use among older outpatients. This study aimed to evaluate the prevalence and risk factors of PIM use in older Chinese outpatients using the 2017 Chinese criteria, 2019 AGS/Beers criteria, and 2014 STOPP criteria, and to assess the concordance among these three criteria. **Methods**: A cross-sectional study was conducted using outpatient prescription data from 59 hospitals across six Chinese cities between January and December 2021. Prescriptions for patients aged ≥65 years were included. PIMs were identified using the three criteria. Concordance was evaluated using weighted kappa statistics. Multivariate logistic regression was used to identify factors associated with PIM use. **Results**: Among 131,894 prescriptions, PIM prevalence was 29.00%, 27.77%, and 19.85% according to the Chinese, Beers, and STOPP criteria, respectively. The most common PIM was estazolam. Concordance was moderate between the Chinese and Beers criteria (kappa = 0.58), STOPP criteria (kappa = 0.625), and good between Beers and STOPP criteria (kappa = 0.73). Factors associated with PIM use included age ≥80 years, polypharmacy, full self-payment, and diagnoses such as sleep disorders and arthritis. **Conclusions**: PIM use is prevalent among older outpatients in China, with varying rates across different criteria and regions. Interventions targeting high-risk groups and specific drug classes may improve medication safety in this population.

## 1. Introduction

By the end of 2021, China’s population aged 65 and older reached 191 million, representing 13.5% of the total population and accounting for one in four older adults globally. This rapid demographic shift brings a significant and growing burden of disease. On average, older adults in China live with health conditions for over eight years. More than 190 million older Chinese individuals have chronic diseases, with 75% of the elderly population managing at least one chronic condition [1]. The treatment of chronic diseases in this population is often centered on a single-disease framework. However, as the number of comorbidities increases, older patients frequently require multiple medications simultaneously, a situation defined as polypharmacy when five or more drugs are used concurrently [2].

Advancing age is associated with pharmacokinetic and pharmacodynamic changes that elevate the risk of drug–drug and drug–disease interactions. When a medication’s potential for harm outweighs its expected benefits, it is classified as a potentially inappropriate medication (PIM) [3]. Adverse drug reactions (ADRs) resulting from PIMs are linked to severe clinical outcomes, including increased mortality, fractures, falls, and hospital readmissions. PIMs are the third leading cause of hospitalization among older adults and the primary cause of hospital-acquired conditions, significantly diminishing their quality of life [4,5,6,7].

Inpatient medication management for older adults is typically under the close supervision of a healthcare team, which allows for real-time monitoring and dynamic adjustments to treatment regimens. In contrast, the long-term use of medications by older outpatients is largely self-managed or overseen by caregivers, without the direct, continuous support of healthcare professionals. This lack of oversight increases the likelihood of medication errors, such as incorrect or missed doses, which in turn elevates the risk of PIMs [8]. Studies have shown that the mortality risk associated with PIMs is higher in older outpatients (50 per 1000) compared to inpatients (39 per 1000) [9,10].

Outpatient clinics are the primary source of chronic disease medications for the older population in China. Given the vast number of older adults requiring long-term pharmacotherapy, the potential for harm from inappropriate medication use is substantial. As the first point of contact for many patients, outpatient settings offer a critical opportunity to identify and rectify PIMs. Doing so can prevent unnecessary drug expenditures and reduce costs associated with additional tests, treatments, and hospitalizations caused by PIMs. Consequently, focusing on medication safety management for older outpatients can yield significant public health and economic benefits by improving the efficiency of healthcare resource allocation [11].

To promote rational medication use and control PIMs, various assessment tools have been developed worldwide. A review of 46 global PIM assessment criteria identified three main types: explicit, implicit, and mixed criteria [12]. Explicit criteria are evidence-based or consensus-based lists of medications to be avoided in certain clinical situations. They are clearly defined, easy to apply without extensive clinical experience, and yield consistent results across different evaluators. Implicit criteria, on the other hand, require a clinician’s judgment based on a comprehensive understanding of the patient’s condition and medication history, making them more time-consuming and less consistent. Due to their practicality and reliability, explicit criteria are more widely used. Our previous research indicates that the most commonly applied PIM criteria for older outpatients in China [13] are the 2019 Beers Criteria [14] and the 2014 STOPP/START Criteria [15]. In 2018, China also released its own set of explicit criteria, the Chinese Criteria for Potentially Inappropriate Medication Use in Older Adults [16].

Despite the availability of these tools, significant research gaps remain. First, most studies on PIMs in older Chinese outpatients have been limited by small sample sizes and a focus on single institutions or regions, which compromises the generalizability of their findings. Second, while some studies have compared PIM rates using different criteria, they have often been restricted to specific disease populations or healthcare settings, limiting their external validity [17]. Therefore, this study was designed as a large-scale, multi-center cross-sectional investigation to determine the prevalence of PIMs in older Chinese outpatients, analyze the sensitivity of the three major explicit criteria, and identify key risk factors. The findings are intended to inform evidence-based policies for rational medication use, guide medical insurance strategies, and support the implementation of national healthy aging initiatives.

## 2. Methods

### 2.1. Study Population, Setting and Data Source

**Figure 1:**
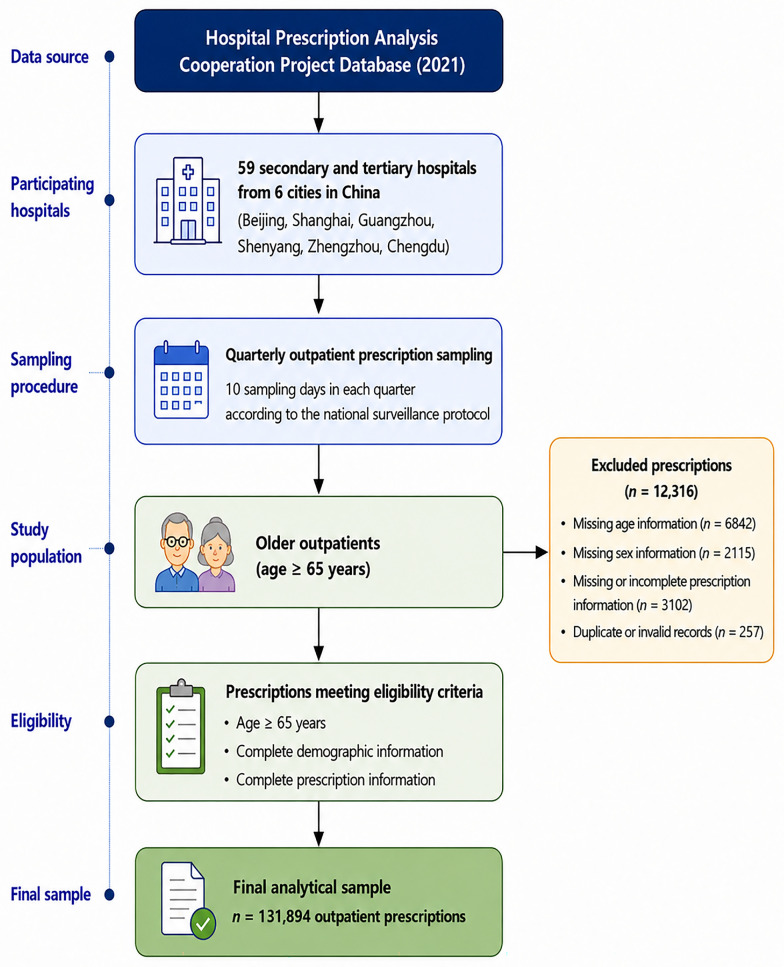
Flowchart of outpatient prescription selection.

This study was based on data from the Hospital Prescription Analysis Cooperation Project, a nationwide prescription surveillance program in China. The database included outpatient prescriptions collected from secondary and tertiary hospitals located in six major cities: Beijing, Shanghai, Guangzhou, Shenyang, Zhengzhou, and Chengdu. Participating hospitals were selected using purposive sampling. Within each hospital, outpatient prescriptions for older adults were randomly sampled on 10 days per quarter, divided into two separate five-day sampling periods. This sampling strategy was designed to capture seasonal variation in prescribing practices while maintaining a standardized surveillance protocol across all participating centers. Prescription data were obtained directly from the Hospital Information System (HIS) whenever electronic medical records were available. For hospitals without a complete HIS, paper prescriptions were manually entered into an Epidata database before being incorporated into the centralized prescription database. All eligible prescriptions from geriatric outpatient clinics collected during the study period were included in the analysis. Because this investigation represented a secondary analysis of an existing nationwide database, no a priori sample size calculation was performed. Instead, the study population consisted of all eligible outpatient prescriptions available between 1 January and 31 December 2021, and the final sample size was therefore determined by data availability rather than statistical power considerations. The database contained information on patients’ demographic characteristics (age, sex, and payment method), prescribing institution (city and department), clinical diagnoses, and detailed medication information, including drug name, dosage form, route of administration, prescribed dose, dosing frequency, and quantity dispensed. Prescriptions issued for individuals aged 65 years or older were eligible for inclusion, whereas records containing only non-therapeutic items (e.g., sterile water for injection or contrast agents used for diagnostic imaging) were excluded. This study was reported in accordance with the Strengthening the Reporting of Observational Studies in Epidemiology (STROBE) statement [18]. The process used to identify the final analytical sample is presented in Figure 1.

### 2.2. Diagnosis and Medication Classification

Disease diagnoses were coded according to the International Classification of Diseases, 10th Revision (ICD-10). Medications were classified based on the Anatomical Therapeutic Chemical (ATC) classification system of the World Health Organization (WHO), using their generic names. A clinician and a clinical pharmacist were responsible for coding the diagnoses. If a diagnosis could not be directly mapped to an ICD-10 code, the clinician assigned a code based on professional judgment. In cases of ambiguity, a panel of two clinicians and one clinical pharmacist made the final decision. For prescriptions with missing diagnostic information, the research team contacted the respective medical institution for clarification. Two clinical pharmacists independently classified all medications. Discrepancies were resolved by a third, senior clinical pharmacist. For prescriptions with missing drug names, the institutions were contacted for verification.

### 2.3. Evaluation Criteria

PIMs were identified using three explicit prescribing tools: the 2017 Chinese Criteria, the 2019 Beers Criteria, and Version 2 of the STOPP Criteria (2014). The 2014 STOPP Criteria were selected to maintain methodological consistency with previous nationwide studies conducted in China, thereby facilitating direct comparison of PIM prevalence across studies. All assessments were restricted to older outpatients and did not include prescriptions issued in palliative or hospice care settings. PIM assessments were performed independently by two clinical pharmacists experienced in chronic disease management using a dedicated electronic assessment platform. The evaluation results were subsequently reviewed by a senior clinical pharmacist specializing in geriatric pharmacotherapy. Any discrepancies identified during the independent assessments were resolved through discussion until consensus was achieved among the three pharmacists.

### 2.4. Statistical Analysis

Categorical variables are presented as frequencies and percentages and were compared using the chi-square test. Continuous variables with a normal distribution are expressed as the mean ± standard deviation (SD), whereas non-normally distributed variables are reported as the median and interquartile range (IQR). Multivariable logistic regression analysis was performed to identify factors independently associated with PIM use. Variables included in the multivariable model were selected *a priori* based on clinical relevance and evidence from previous studies rather than automated variable selection procedures. The results are presented as odds ratios (ORs) with corresponding 95% confidence intervals (CIs). Agreement among the 2017 Chinese Criteria, the 2019 AGS Beers Criteria, and the 2014 STOPP Criteria was evaluated using Cohen’s kappa coefficient, which is appropriate for assessing agreement between dichotomous classifications. Kappa values > 0.70 were interpreted as indicating good agreement, values between 0.40 and 0.70 as moderate agreement, and values < 0.40 as poor agreement [19]. All statistical analyses were conducted using IBM SPSS Statistics version 26.0 (IBM Corp., Armonk, NY, USA). A two-sided *p* value < 0.05 was considered statistically significant.

## 3. Results

### 3.1. Basic Characteristics of Patients

This study analyzed 131,894 outpatient prescriptions for older adults collected from 59 medical institutions across six cities (Beijing, Chengdu, Guangzhou, Shanghai, Shenyang, and Zhengzhou) in 2021, after excluding prescriptions for solvents, contrast agents, and other non-medicinal components. The overall prevalence of PIMs, as assessed by the Chinese criteria, was 29.00% (38,245 PIM prescriptions). Regional variations were observed, with Guangzhou exhibiting the highest PIM rate (41.72%) and Shenyang the lowest (21.60%). When assessed by the Beers criteria, the overall PIM rate was 27.77% (36,621 PIM prescriptions). For the STOPP criteria, the rate was 19.85% (26,176 PIM prescriptions). Consistent with the Chinese criteria, Guangzhou (36.29%) and Zhengzhou (30.64%) showed the highest PIM rates according to the Beers criteria and STOPP criteria, respectively, while Shenyang consistently had the lowest rates (20.52% and 15.52%). Across all three criteria, PIM rates were higher in older patients aged ≥80 years. A statistically significant increase in PIM rates was observed with an increasing number of diagnoses and medications. Hypertension was the most common prescription diagnosis, while sleep disorders were associated with the highest PIM rates (Table 1).

### 3.2. Concordance Between the Three Criteria

The concordance between the Chinese criteria and the Beers criteria was moderate (κ = 0.58). The Chinese criteria showed moderate concordance with the STOPP criteria (κ = 0.625). A good concordance was observed between the Beers criteria and the STOPP criteria (κ = 0.73) (Table 2).

### 3.3. Prevalence of PIMs and the Most Frequent PIMs

The Chinese criteria comprise 106 relevant standards, of which 71 (66.98%) were applicable in this study. Application of the Chinese criteria identified 49,394 PIM instances within the 38,245 PIM prescriptions, with the majority (79.01%) containing only one PIM. The Beers criteria include 90 relevant standards, with 46 (51.11%) being applicable in this study. These criteria identified 48,895 PIM instances across 36,621 PIM prescriptions, with 77.10% containing a single PIM. The STOPP criteria are structured into 13 categories, encompassing 81 standards, of which 38 (46.91%) were relevant to this study. These criteria detected 30,843 PIM instances in 26,176 PIM prescriptions, with 85.75% involving a single PIM (Table 3).

According to the Chinese criteria, the five most frequently detected PIMs were clopidogrel (8893, 23.25%), estazolam (6643, 17.37%), zolpidem (4510, 11.79%), insulin (4253, 11.12%), and alprazolam (3606, 9.43%). Based on the Beers criteria, the top five were estazolam (6643, 18.14%), hydrochlorothiazide (4724, 12.90%), zolpidem (4510, 12.32%), alprazolam (3606, 9.85%), and flurbiprofen (2397, 6.55%). The STOPP criteria identified estazolam (6643, 25.34%), zolpidem (4510, 17.23%), alprazolam (3606, 13.78%), zopiclone (2529, 9.66%), and furosemide (1110, 4.24%) as the five most common PIMs (Table 4).

### 3.4. Factors Associated with PIM Use

**Figure 2:**
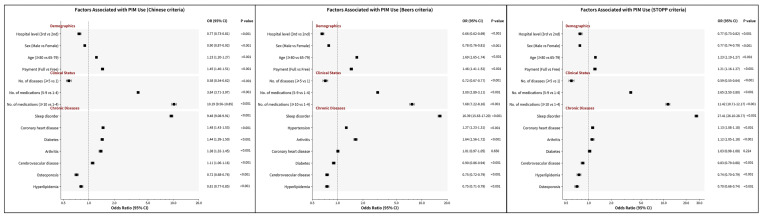
Multivariate logistic regression analysis of factors associated with PIM use.

Multicollinearity was assessed using variance inflation factors (VIFs), and no evidence of problematic multicollinearity was observed (all VIFs < 5). Multivariate logistic regression analysis, using PIM use (non-PIM = 0, PIM = 1) as the dependent variable, revealed several significant factors associated with PIM use across all three criteria. Age ≥80 years was positively associated with PIM use (OR: 1.232 by Chinese criteria, OR: 1.694 by Beers criteria, OR: 1.233 by STOPP criteria). Polypharmacy (more medications) also significantly increased the likelihood of PIMs (OR: 3.836 and 10.186 for Chinese criteria, OR: 2.988 and 7.676 for Beers criteria, OR: 3.645 and 11.419 for STOPP criteria). Full self-payment for prescriptions was another identified factor associated with (OR: 1.453 by Chinese criteria, OR: 1.459 by Beers criteria, OR: 1.213 by STOPP criteria). Patients with diagnoses such as sleep disorders (OR: 9.484 by Chinese criteria, OR: 16.394 by Beers criteria, OR: 27.406 by STOPP criteria) and arthritis (OR: 1.384 by Chinese criteria, OR: 1.637 by Beers criteria, OR: 1.118 by STOPP criteria) were also found to be more likely to have PIMs (Figure 2).

## 4. Discussion

This study analyzed 131,894 outpatient prescriptions for older adults from 59 medical institutions across six Chinese cities in 2021, revealing a PIM rate of 29.00% according to the Chinese criteria. This prevalence is higher than the 15.81% reported in a 2014 study on PIM rates in outpatient and emergency departments across six Chinese cities [20]. The discrepancy can be attributed to two main factors: the earlier study utilized only a subset of the Chinese criteria, and its study population was not exclusively limited to prescriptions from geriatric departments. Our findings suggest that PIMs are more common in prescriptions from geriatric departments. Patients attending geriatric clinics often present with a higher burden of comorbidities and geriatric syndromes, and their primary need is typically for chronic disease management [4,5]. This predisposes them to a greater risk of polypharmacy and PIMs [21,22]. It should be noted that the applicability of the three explicit prescribing criteria differed because several Beers and STOPP indicators require detailed clinical information that was unavailable in the present prescription database. As a result, the reported prevalence of PIMs identified by these criteria may have been underestimated. Therefore, differences observed between the three criteria should be interpreted with caution, as they may partly reflect differences in data availability rather than intrinsic differences in the performance of the prescribing tools. Although the present study included prescriptions from 59 secondary and tertiary hospitals located in six major cities across China, participating hospitals were selected using purposive sampling rather than probability-based sampling. Therefore, our findings primarily reflect prescribing patterns in large urban medical institutions and should not be considered fully representative of all outpatient settings nationwide. In particular, prescribing practices may differ in primary healthcare institutions, rural hospitals, community healthcare centers, and long-term care facilities. Nevertheless, the multicenter design, large sample size, and standardized data collection procedures provide valuable insights into prescribing patterns among older outpatients attending major hospitals in China.

Regional variations in PIM rates were observed across different cities when assessed by the Chinese criteria. Compared to PIM rates for specific diseases, the PIM rate for older patients with chronic diseases, not limited to a single specialty, was lower when assessed by the Chinese criteria [17]. This suggests that targeted interventions focusing on specific diseases might be more effective in reducing PIMs. When assessed by the Beers criteria, the PIM rate for older Chinese outpatients was 27.77%, which is lower than Japan’s PIM rate of 43.70% [23] but higher than South Korea’s outpatient PIM rate of 19.32% [24]. Compared to prescriptions for older Chinese patients with Alzheimer’s disease [25], the PIM use rate in older patients with chronic diseases was lower. This difference might be explained by the complex medication regimens often required for Alzheimer’s patients, who frequently present with psychiatric symptoms in addition to cognitive decline, leading to the use of anti-dementia drugs, antipsychotics, and sedative-hypnotics. Furthermore, adverse clinical outcomes in Alzheimer’s patients are often linked to PIMs, and these outcomes can further exacerbate PIM issues [26,27].

The PIM rate for older Chinese outpatients, as assessed by the STOPP criteria, was 19.85%, which is lower than Bahrain’s PIM rate of 34.07% [28] but higher than South Korea’s outpatient PIM rate of 1.26% [29]. The lower detection rate by the STOPP criteria, compared to the Chinese and American criteria, may be due to its requirement for a comprehensive understanding of the patient’s entire medication history, clinical information, and laboratory results, which are often incomplete in outpatient prescription data. The European PIM assessment criteria comprise 81 standards across 13 categories, but only 38 (46.91%) were applicable in this study, further contributing to the lower detection rate.

To our knowledge, this is the first study to evaluate the concordance of three major PIM assessment tools (Chinese criteria, Beers criteria, and STOPP criteria) in prescriptions for older Chinese outpatients. Despite being developed based on different populations and research data, these criteria are widely used in older Chinese patients. Our study found moderate concordance between the Chinese criteria and both the American and STOPP criteria, and good concordance between the American and STOPP criteria. This finding differs slightly from another study on older hospitalized patients in China [30]. Additionally, a study in Portugal on hospitalized patients aged 65 or older reported poor concordance among the Beers criteria, STOPP criteria, and the EU(7)-PIM list [31]. Although the kappa coefficients indicated moderate agreement according to conventional statistical benchmarks, the observed values (approximately 0.58–0.63) also suggest substantial discrepancies in the identification of potentially inappropriate medications. In other words, a considerable proportion of prescriptions were classified differently depending on the explicit prescribing criterion applied. These findings indicate that the three criteria are not interchangeable in routine clinical practice, and that the estimated prevalence of PIM use, as well as the patients identified for medication review, may vary according to the assessment tool selected. From a clinical perspective, these differences may influence opportunities for medication review, deprescribing interventions, and quality improvement initiatives. Therefore, healthcare professionals should recognize that each criterion reflects different prescribing priorities and should interpret the results within the clinical context rather than relying exclusively on a single explicit prescribing tool. The observed differences in prevalence and concordance indicate that each criterion captures different aspects of potentially inappropriate prescribing because they were developed using different evidence bases, expert consensus processes, and healthcare settings. Therefore, these criteria should be regarded as complementary rather than interchangeable when evaluating prescribing quality in older adults. Because no longitudinal clinical outcome data were available in this study, our findings should not be interpreted as demonstrating the clinical superiority of any individual criterion. Future prospective studies linking PIM identification to adverse drug reactions, falls, hospitalization, fractures, and mortality are warranted to determine which criterion provides the greatest clinical utility in older Chinese adults. For example, benzodiazepines and Z-drugs were consistently identified among the most common PIMs across all three criteria, suggesting broad consensus regarding their potential risks in older adults. In contrast, certain medications, such as clopidogrel, were identified primarily by the Chinese criteria, highlighting important differences in medication coverage. These observations may help prioritize medications or therapeutic classes for future evaluation when updating the Chinese PIM criteria. However, decisions regarding whether specific medications should be retained, modified, or excluded from future versions of the Chinese criteria require prospective outcome studies, pharmacoepidemiologic evidence from Chinese populations, and multidisciplinary expert consensus, rather than concordance analyses alone.

One important contributor to the higher prevalence of PIMs identified by the Chinese criteria was the inclusion of clopidogrel as a potentially inappropriate medication under specific circumstances. Clopidogrel accounted for a substantial proportion of all PIMs identified in the present study. This recommendation reflects concerns regarding bleeding risk in older adults, particularly among patients with characteristics that increase susceptibility to hemorrhagic complications. In contrast, the Beers Criteria and the STOPP Criteria generally evaluate antiplatelet therapy according to specific clinical indications and treatment contexts rather than classifying clopidogrel itself as a potentially inappropriate medication. Consequently, differences in the underlying development principles of these explicit prescribing criteria contribute substantially to the observed variation in PIM prevalence and only moderate agreement between the three tools. The most common class of drugs identified as PIMs by all three criteria was benzodiazepines, a finding consistent with most previous studies [32,33,34]. The high prescription rate of benzodiazepines can be attributed to the high prevalence of insomnia in older adults. Over 80% of patients were prescribed medication for insomnia, yet most prescribed benzodiazepines and sedative-hypnotics are inappropriate for older patients [35]. Due to age-related physiological changes, older adults are more sensitive to medications. Despite the known association of benzodiazepines with increased risks of cognitive impairment, falls, and fractures, their use remains common in this population [36]. Current guidelines recommend non-pharmacological therapies, such as stimulus control, sleep restriction, relaxation training, and cognitive behavioral therapy, as first-line treatments for insomnia in older patients [37,38]. A review further emphasized that cognitive behavioral therapy is the preferred initial treatment for sleep disorder, and benzodiazepines, especially for long-term use, are not recommended for older adults [39]. While Z-drugs are often considered first-line agents for insomnia due to their pharmacokinetic and safety profiles compared to benzodiazepines, research indicates they carry similar risks of ADRs, including impaired balance and increased fall risk in older adults [40]. A study comparing zolpidem and benzodiazepines found that zolpidem was associated with a higher risk of fractures compared to alprazolam and lorazepam [41]. Interestingly, clopidogrel was the most frequently detected PIM when assessed by the Chinese criteria.

Our study also revealed that the PIM rate in outpatient prescriptions for older patients aged 80 years and above was higher than for those aged 65–79 years. As individuals age, their physiological functions and immune responses decline, leading to an increased prevalence of comorbidities [30]. For instance, studies have reported that 11.1% of individuals in South China have comorbidities, with the prevalence reaching 47.5% in older adults; in North China, 24.7% of the population has comorbidities, with 56.6% of older women affected [31]. The comorbidity rate generally increases with age. The presence of multiple comorbidities often necessitates long-term use of various medications, which elevates the risk of PIMs. The inverse association observed between the number of chronic diseases and PIM use after multivariable adjustment should be interpreted cautiously. Because the model simultaneously adjusted for the number of medications, which is closely correlated with multimorbidity, this finding may reflect the influence of collinearity or over-adjustment rather than a protective effect of multimorbidity itself. Furthermore, the concurrent use of multiple drugs can lead to drug-induced diseases [32], requiring additional medications and further escalating the PIM risk. Research consistently shows that polypharmacy is an independent factor associated with PIMs, with each additional medication in a PIM assessment increasing the patient’s exposure risk by 5.2% [42]. We also observed that full self-payment was independently associated with a higher likelihood of PIM use across all three criteria. The mechanisms underlying this association remain uncertain. One possible explanation is that differences in reimbursement policies may influence prescribing behavior and medication accessibility. Alternatively, payment status may reflect other unmeasured factors, such as patient preferences, healthcare utilization, socioeconomic status, or prescribing practices, which were not available in our database. Therefore, this finding should be interpreted cautiously and requires confirmation in future studies incorporating more detailed clinical and prescribing information.

From a clinical perspective, our findings suggest that explicit PIM criteria should be viewed as complementary rather than interchangeable tools in outpatient medication review. While medications consistently identified across multiple criteria may represent higher-priority targets for medication optimization, criterion-specific medications should be further evaluated using real-world outcome data before recommendations are incorporated into future revisions of the Chinese criteria. Future prospective studies integrating prescribing data with adverse drug reactions, falls, hospitalization, and mortality will be essential to establish the comparative clinical utility of different PIM criteria in older Chinese adults.

Several limitations should be acknowledged. First, the unit of analysis in this study was individual outpatient prescriptions rather than unique patients. Because the anonymized database did not include patient identifiers, repeated prescriptions from the same individual could not be identified. Consequently, the assumption of independence between observations may have been violated, potentially influencing the estimated prevalence of PIM use and the precision of the regression estimates. Future studies based on longitudinal patient-level databases should account for repeated observations using appropriate statistical methods, such as generalized estimating equations or mixed-effects models. Second, although the Hospital Prescription Analysis Cooperation Project database contained comprehensive prescription information, it lacked several important patient-specific clinical variables required for the complete application of some AGS Beers and STOPP criteria, including therapeutic indications, medication dosage, treatment duration, renal function, laboratory test results, disease severity, and other relevant clinical characteristics. Consequently, only those prescribing criteria that could be reliably evaluated using the available data were applied. This limitation may have led to an underestimation of the prevalence of PIMs identified by the AGS Beers and STOPP criteria and may also have affected comparisons of prevalence estimates and agreement among the three explicit prescribing tools. More importantly, because medication appropriateness cannot be fully evaluated without comprehensive clinical information, the present study should be interpreted as an assessment of potentially inappropriate prescribing based on available prescription records rather than a comprehensive evaluation of overall medication appropriateness. Future studies integrating prescription data with detailed clinical information and laboratory results are warranted to enable a more complete assessment of prescribing quality. Third, this was a cross-sectional study based on outpatient prescription records. Therefore, although several demographic and clinical characteristics were found to be associated with PIM use, causal relationships cannot be inferred because the temporal sequence between exposure and outcome could not be established. In addition, although the multivariable analyses adjusted for several important demographic and clinical characteristics, residual confounding cannot be excluded because information on renal function, frailty, cognitive impairment, educational level, socioeconomic status, physician specialty, hospital characteristics, and other potentially important variables was unavailable. Consequently, the observed associations should be interpreted with appropriate caution. Furthermore, the database did not include longitudinal follow-up or outcome data. Consequently, although the three criteria differed in the prevalence of PIMs identified and their level of agreement, we were unable to determine which criterion better predicted clinically relevant outcomes, such as adverse drug reactions, falls, fractures, hospitalization, or mortality. Therefore, the present study should be interpreted as a comparison of prescribing assessment tools rather than a validation of their clinical performance. Finally, the study population was limited to older adults attending outpatient clinics in secondary and tertiary hospitals. Therefore, our findings may not be generalizable to older adults receiving care in primary healthcare institutions, community healthcare centers, nursing homes, or other long-term care settings.

## 5. Conclusions

This study compared the use of three explicit prescribing criteria for identifying PIMs among older outpatients in China. The Chinese Criteria, Beers Criteria, and STOPP Criteria identified substantially different proportions of PIMs and demonstrated only moderate agreement, indicating that they capture different aspects of potentially inappropriate prescribing rather than being clinically interchangeable. These findings provide important evidence for understanding the variability in PIM assessment and may help inform medication review strategies for older adults in China. However, because of the cross-sectional study design and the absence of longitudinal outcome data, the observed associations should be interpreted with caution. Future prospective studies incorporating comprehensive clinical information and clinically relevant outcomes are warranted to validate these findings and determine the comparative clinical utility of different PIM criteria in older Chinese adults.

## Figures and Tables

**Table 1:** Basic characteristics of older outpatients.

Characteristics	Total	Chinese Criteria, PIM (n, %)	Beers Criteria, PIM (n, %)	STOPP Criteria, PIM (n, %)
Total (%)	131,894	38,245 (29.00)	36,621 (27.77)	26,176 (19.85)
City				
Beijing	18,429	6637 (36.01)	6609(35.86)	4576(24.83)
Chengdu	8543	3056 (35.77)	2734 (32.00)	1766 (20.67)
Guangzhou	17,737	7400 (41.72)	6436 (36.29	4859 (27.39)
Shanghai	44,176	11,084 (25.09)	11,405 (25.82)	7711 (17.46)
Shenyang	39,115	8449 (21.60)	8026 (20.52)	6071 (15.52)
Zhengzhou	3894	1619 (41.58)	1411 (36.24)	1193 (30.64)
Hospital level				
2nd	10,314	3013 (29.21)	3181 (30.84)	2057 (19.94)
3rd	121,580	35,232 (28.98)	33,440 (27.50)	24,119 (19.84)
Sex, n (%)				
Male	83,369	24,035 (28.83)	22,132 (26.55)	15,789 (18.94)
Female	48,525	14,210 (29.28)	14,489 (29.86)	10,387 (21.41)
Age, years (IQR), n (%)	80 (71, 88)			
65–79	63,893	16,100 (25.20)	14,079 (22.04)	10,879 (17.03)
≥80	68,001	22,145 (32.57)	22,542 (33.15)	15,297 (22.50)
No. of diseases [IQR]	1 [1, 3]			
1	81,696	19,098 (23.38)	18,943 (23.19)	13,056 (15.98)
2–4	33,312	11,455 (34.39)	10,550 (31.67)	7611 (22.85)
≥5	16,886	7692 (45.55)	7128 (42.21)	5509 (32.62)
No. of medications [IQR], n (%)	2 [2, 4]			
1–4	103,398	22,426 (21.69)	22,958 (22.20)	15,420 (14.91)
5–9	22,008	11,153 (50.68)	9588 (43.57)	7197 (32.70)
≥10	6488	4666 (71.92)	4075 (62.81)	3559 (54.86)
Quarter of visit				
Q1	33,656	9989 (29.68)	9696 (28.81)	6948 (20.64)
Q2	34,041	9789 (28.76)	9364 (27.51)	6663 (19.57)
Q3	31,190	8952 (28.70)	8448 (27.09)	6068 (19.45)
Q4	33,007	9515 (28.83)	9113 (27.61)	6497 (19.68)
Payment				
Free	54,134	14,241 (26.31)	13,603 (25.13)	10,488 (19.37)
Partial Fee	50,893	14,920 (29.32)	14,310 (28.12)	9554 (18.77)
Full fee	26,867	9084 (33.81)	8708 (32.41)	6134 (22.83)
Type of chronic disease, n (%)				
Hypertension	40,977	14,015 (34.20)	13,845 (33.79)	9387 (22.91)
Coronary heart disease	29,532	11,969 (40.53)	9978 (33.79)	7605 (25.75)
Diabetes	18,146	7254 (39.98)	5524 (30.44)	4284 (23.61)
Cerebrovascular disease	15,284	5522 (36.13)	4485 (29.34)	3379 (22.11)
Sleep disorder	13,572	9707 (71.52)	10,745 (79.17)	10,254 (75.55)
Arthritis	13,218	5250 (39.72)	5169 (39.11)	3397 (25.70)
Hyperlipidemia	13,086	4791 (36.61)	4249 (32.47)	3170 (24.22)
Osteoporosis	11,767	3797 (32.27)	3490 (29.66)	2587 (21.99)
Chronic gastritis	6895	1976 (28.66)	1758 (25.50)	1333 (19.33)
Prostatic hyperplasia	6460	2583 (39.98	2207 (34.16)	1621 (25.09)

PIM, potentially inappropriate medication; IQR, interquartile range; CNY, Chinese Yuan.

**Table 2:** Concordance between the Chinese criteria, Beers criteria and STOPP criteria.

Chinese Criteria	Beers Criteria		κ	*p*
	Yes	No		
Yes	26,170	12,075	0.58	<0.001
No	10,451	83,198		
**Beers criteria**	**STOPP criteria**			
	Yes	No		
Yes	24,880	11,741	0.73	<0.001
No	1296	93,977		
**STOPP criteria**	**Chinese criteria**			
	Yes	No		
Yes	22,986	3190	0.625	<0.001
No	15,259	90,459		

**Table 3:** The number of PIMs used by older outpatients.

Characteristics	Chinese Criteria	Beers Criteria	STOPP Criteria
PIM prescription	38,245	36,621	26,176
PIMs, n (%)	49,394	48,895	30,843
1 PIM	30,219 (79.01)	28,234 (77.10)	22,446 (85.75)
2 PIMs	5802 (15.17)	5753 (15.71)	2995 (11.44)
≥3 PIMs	2224 (5.82)	2634 (7.19)	735 (2.81)

PIM, potentially inappropriate medication.

**Table 4:** The five most consumed PIMs used by older outpatients.

Number	Chinese Criteria	N = 38,245 (%)	Beers Criteria	N = 36,621 (%)	STOPP Criteria	N = 26,176 (%)
1	Clopidogrel	8893 (23.25)	Estazolam	6643 (18.14)	Estazolam	6643 (25.34)
2	Estazolam	6643 (17.37)	Hydrochlorothiazide	4724 (12.90)	Zolpidem	4510 (17.23)
3	Zolpidem	4510 (11.79)	Zolpidem	4510 (12.32)	Alprazolam	3606 (13.78)
4	Sliding Scale Insulin	4253 (11.12)	Alprazolam	3606 (9.85)	Zopiclone	2529 (9.66)
5	Alprazolam	3606 (9.43)	Flurbiprofen	2397 (6.55)	Furosemide	1110 (4.24)

## Data Availability

The data presented in this study are available on request from the corresponding author due to compliance with ethical requirements and protection of participant privacy. All relevant data will be shared with other researchers who send formal requests to the corresponding author. Please let us know if further information is needed.
